# Assessment of Pupillary Light Reflex Alterations in Pediatric Diabetic Ketoacidosis-Induced Encephalopathy: A Retrospective Analysis Using Quantitative Pupillometry

**DOI:** 10.7759/cureus.84284

**Published:** 2025-05-17

**Authors:** Elber Y Aydin, Matthew Garber, Jose Irazuzta

**Affiliations:** 1 Pediatrics, University of Florida College of Medicine – Jacksonville, Jacksonville, USA; 2 Hospital Medicine, University of Florida College of Medicine – Jacksonville, Jacksonville, USA; 3 Pediatric Critical Care, University of Florida College of Medicine – Jacksonville, Jacksonville, USA

**Keywords:** diabetic ketoacidosis, dka, multimodal neuromonitoring, neurocritical care, pediatric encephalopathy, pupillometry

## Abstract

Diabetic ketoacidosis (DKA) encephalopathy (DKAe) and its associated cerebral edema are serious complications of DKA. This study aimed to use quantitative pupillometry to determine whether changes in pupillary response are associated with alterations in neurological status in pediatric patients admitted to the pediatric intensive care unit (PICU) with DKA. Conducted as a retrospective electronic medical record review at Wolfson Children’s Hospital in Jacksonville, Florida, the study included 21 pediatric patients, between 6 and 17 years of age, diagnosed with DKA. These patients were divided into two groups based on the presence or absence of encephalopathy at admission. Pupillometry readings were obtained at two time points: at admission (T0) and after the resolution of both DKA and DKAe (T1).

At T0, the constriction velocity (CV) and maximum constriction velocity (MCV) were significantly lower in patients with encephalopathy compared to those without. By T1, there was no significant difference between the two groups, though the encephalopathic group demonstrated a significant improvement in pupillary velocities over time, while the non-encephalopathic group did not. The median time between measurements was 12.5 hours (IQR: 10-17) in the encephalopathic group and 10 hours (IQR: 10-11) in the non-encephalopathic group. The area under the curve for CV and MCV of the right eye at admission was 0.864 (95% CI: 0.709-1.0) and 0.845 (95% CI: 0.657-1.0), respectively. The receiver operating characteristic (ROC) curves were generated using SPSS (Statistical Package for the Social Sciences), with slower velocities considered evidence for encephalopathy. The best cutoff for CV was 3.26 mm/s, yielding a sensitivity of 100% and specificity of 54.5%, while the best cutoff for MCV was 4.545 mm/s, with a sensitivity of 90% and specificity of 81.8%. These findings suggest that DKAe leads to a transient slowing of CV and MCV, likely reflecting a temporary alteration in the parasympathetic component of the pupillary light reflex, which resolves as encephalopathy subsides. This raises important questions about the role of autonomic nervous system dysfunction in the pathophysiology of DKAe and highlights the potential utility of pupillometry as a noninvasive tool for monitoring neurological status in pediatric patients with DKA.

## Introduction

Diabetic ketoacidosis (DKA) encephalopathy (DKAe) and associated cerebral edema have an incompletely elucidated, complex pathobiology. While cerebral edema resulting in mortality and long-term morbidity is a rare complication of severe DKA with an incidence of approximately 1%, children with DKA are known to have subclinical brain edema [1].

DKA causes transient changes in brain volume and diffusivity on imaging, particularly affecting the cerebral white matter in the frontal lobes [2]. These changes can result in persistent impairments in attention and memory [2]. Additionally, recurrent episodes of DKA are associated with lower global cognitive function, with the strongest correlation seen in individuals with childhood-onset diabetes [3].

Increased blood-brain barrier (BBB) transcellular permeability and disruption of tight junctions leading to extravasation of albumin secondary to an inflammatory cascade is one of the putative mechanisms of DKA-associated cerebral edema confirmed by brain MRI [4]. Another factor, as shown by Hoffman et al., is a period of cerebral loss of autoregulation and resulting vasoplegia, which may contribute to vasogenic edema [5].

Patients with DKA admitted to the pediatric intensive care unit (PICU) require frequent neurological assessments, including clinical examinations such as the Glasgow Coma Scale (GCS) and evaluation of pupil reactivity. The application of GCS in pediatric populations poses significant challenges, with studies assessing its reliability yielding heterogeneous results [6]. Several modifications to the scoring system have been proposed to improve its accuracy in the pediatric population [7-9].

Clinical bedside assessment of pupil reactivity is highly dependent on the observer and has a large interobserver variability [10]. Quantitative pupillometry is an objective, noninvasive technique that measures the changes in pupillary size and response to light. Quantitative pupillometry provides a granular assessment allowing separate analysis of the sympathetic and parasympathetic response, which cannot be evaluated by clinical examination. Quantitative pupillometry correlates with intracranial pressure changes and has been used in the evaluation of midline shifts in traumatic brain injury, as well as to serve as an early predictor tool after cardiac arrest in several studies [11-13]. Pupillometry has greater reliability and accuracy than the traditional penlight method [10,14].

Pupil size and reactivity are controlled by the interplay between the parasympathetic and sympathetic nervous systems. Alterations in autonomic response have been documented in traumatic brain injury, with a decrease in heart rate variability linked to poorer outcomes [15]. The effects of brain edema and DKAe on pupillary reflexes in pediatric patients are not well known.

In this study, we aimed to use quantitative pupillometry to assess whether changes in pupillary response to light are associated with alterations in neurological status in patients admitted to the PICU with DKA [16].

## Materials and methods

This study used a convenience sample of patients with DKA admitted to the PICU at Wolfson Children’s Hospital, Jacksonville, between July 1, 2021, and October 31, 2023. A retrospective chart review was conducted, and demographic data, the presence of encephalopathy, and pupillometry findings were collected at admission (T0) and after the resolution of DKA and DKAe (T1) using the electronic medical record systems Cerner and EPIC. Patients receiving medications known to affect the pupillary light reflex, as well as those with pre-existing ocular conditions, were excluded from the study. None of our patients in the patient received 3% saline or mannitol as part of their treatment. Recurrent admissions of the same patients were not included in the study. The presence of DKA was defined as blood glucose > 200 mg/dL, metabolic acidosis (venous pH < 7.3 or serum bicarbonate < 15 mEq/L), and the presence of ketones in the blood (>3 mmol/L beta-hydroxybutyrate) or urine (moderate/large). The presence of encephalopathy was defined as the presence of altered mental status that included disorientation, speech/language impairments, and the inability to follow commands. At the time of PICU admission, the pediatric critical care physician documented the presence or absence of encephalopathy in the electronic medical record, which was used to assign patients to the appropriate groups. DKA resolution was defined as serum bicarbonate levels greater than 15 mmol/L or beta-hydroxybutyrate levels less than 2 mmol/L, along with a well-appearing child who tolerates an oral intake challenge. DKAe resolution was defined as the resolution of DKA, the absence of any symptoms mentioned above, and the child returning to baseline neurological status. The data were collected from the nursing notes, the physician's physical examination notes, and the neurological examination flowsheet in the chart for the pupillary examination readings.

NPi-300 Pupillometer by NeurOptics, Irvine, California, was utilized for pupillary assessment. The assessments were conducted by nurses following the manufacturer’s instructions. The pupillometer measures the Neurological Pupil Index (a proprietary index created by an algorithm that incorporates several variables, combining and comparing them against a mean derived from a reference distribution of healthy study participants), maximum pupil diameter before constriction, the pupil diameter at peak constriction, the percentage change in pupil size from maximum to minimum, the latency of constriction onset following the light stimulus, the average speed of pupil constriction, and the average speed of dilation back to the resting size (dilation velocity (DV)), in addition to the constriction velocity (CV) and maximum constriction velocity (MCV). Two pupillometry readings were performed the first at admission, and the second after the resolution of DKA and DKAe based on the above-mentioned criteria.

The data for CV (the speed at which the pupil changes from baseline to its smallest size over time, or the amount of pupillary constriction divided by the duration of constriction) and MCV (the peak value of velocity during pupillary constriction), measured in millimeters per second (mm/s), were collected from the pupillometry values recorded during the neurological examination in the patients' charts. Both eyes were measured sequentially in a single session, and we elected to include data from only one eye based on several considerations. To minimize variability in pupillometry readings, ambient light conditions in the patient rooms were standardized, with consistent artificial lighting and patient beds positioned away from direct natural light. Measurements were consistently initiated with the right eye by nursing staff, and in the absence of a mandated waiting period between eyes, the left eye may have been influenced by the contralateral pupillary reflex. While significant and nonsignificant findings were consistently mirrored between eyes, variability was greater in the left eye. We attribute this to the contralateral reflex and potentially reduced precision during the second measurement [17,18]. To minimize this source of heterogeneity and simplify reporting, we present data from the right eye only. This study was exempt from review by the Baptist Health and University of Florida Institutional Review Boards (STUDY23-009).

Due to the small sample size and non-normal data distributions, we used non-parametric statistics. CV and MCV of the eyes, measured in millimeters per second (mm/s), were compared between the groups at T0 and T1 using the Mann-Whitney U test. The median difference of CV and MCV between T0 and T1 for each patient’s eye was computed using the Wilcoxon signed rank test. We calculated the time intervals between T0 and T1 for both groups and performed descriptive statistics (median and IQR using Tukey's hinges). Receiver operating characteristic (ROC) curve analysis was performed to evaluate the diagnostic performance of CV and MCV of the right eye at admission. The area under the curve (AUC) with 95% confidence intervals (CI) was calculated for each parameter to determine their predictive accuracy. To account for multiple comparisons, we applied the Bonferroni correction by dividing the conventional alpha level of 0.05 by the four main unpaired comparisons, resulting in an adjusted significance threshold of 0.0125.

## Results

Twenty-one patients were included and divided into two groups based on clinical assessment documented in the electronic health record: 10 with and 11 without encephalopathy at admission. Table 1 shows patient demographics, such as age, gender, encephalopathic status, and diabetes mellitus status (new or known). At T0, the CV and MCV of the right eye were significantly reduced in the encephalopathic group compared to the non-encephalopathic group, with a median difference of -0.95 (95% CI: -1.66, -0.34; p = 0.004) for CV and -1.65 (95% CI: -2.95, -0.51; p = 0.006) for MCV. At T1, the CV and MCV of the right eye were not significantly different, with a median difference of 0.525 (95% CI: -0.07, 1.13; p = 0.085) for CV and 0.285 (95% CI: -0.75, 1.4; p = 0.349) for MCV in the encephalopathic group compared to the non-encephalopathic group.

**Table 1:** Age, gender, presentation, and diabetes mellitus status

Group	Number of Patients	Age (Years) [Median, IQR]	Gender (M/F)	Known/New-Onset Diabetes
All	21	13 [10, 15]	13 Males/8 Females	9 Known/12 New-onset diabetes
Encephalopathic	10	13.5 [9.25, 15]	7 Males/3 Females	6 Known/4 New-onset diabetes
Non-encephalopathic	11	12 [10, 15]	6 Males/5 Females	3 Known/8 New-onset diabetes

Only the encephalopathic group showed a significant difference in velocities between T0 and T1 (see Table 2 for all three comparisons). The encephalopathic group had a median time of 12.5 hours (IQR: 10,17), while the non-encephalopathic group had a median time of 10 hours (IQR: 10,11) between measurements. The AUC for CV and MCV of the right eye at admission was 0.864 (95% CI: 0.709-1.0) and 0.845 (95% CI: 0.657-1.0), respectively. The ROC curves were generated using SPSS (Statistical Package for the Social Sciences) with slower velocities considered evidence for encephalopathy. The best cutoff for CV was 3.26 mm/s with a sensitivity of 100% and specificity of 54.5%. The best cutoff for MCV was 4.545 mm/s with a sensitivity of 90% and a specificity of 81.8%.

**Table 2:** Comparison of constriction velocity and maximum constriction velocity between groups at T0 and T1, with paired comparisons within each group over time Data are displayed as median with interquartile range and [MD (95% CI)] p-values. CV: Constriction velocity; MCV: Maximum constriction velocity; T0: Pupillometry reading at admission; T1: Pupillometry reading after the resolution of DKA; MD: Median difference; CI: Confidence interval.

Group	CV T0 (mm/s)	[MD (95% CI)] p-value	CV T1 (mm/s)	[MD (95% CI)] p-value
Encephalopathic	2.32 (1.37,2.85)	[-0.95 (-1.66,-0.34)] p = 0.004	3.56 (3.20,4.17)	[0.525 (-0.07,1.13)] p = 0.085
Non-encephalopathic	3.31 (2.82,3.53)		2.86 (2.66,3.78)	
	MCV T0 (mm/s)	[MD (95% CI)] p-value	MCV T1 (mm/s)	[MD (95% CI)] p-value
Encephalopathic	3.25 (1.99,4.27)	[-1.65 (-2.95,-0.51)] p = 0.006	5.37 (4.76,6.29)	[0.285 (-0.75,1.4)] p = 0.349
Non-encephalopathic	5.23 (4.68, 5.83)		5.14 (4.02,6.02)	
		CV T0-T1		MCV T0-T1
Encephalopathic		[1.47 (0.91,2.17)] p = 0.005		[2.3 (1.12,3.51)] p = 0.005
Non-encephalopathic		[-0.05 (-0.41,0.37)] p = 0.722		[0.057 (-0.43,0.73)] p = 0.79

## Discussion

In this study, we focused on CV and MCV after our initial findings showed that these variables had discriminatory values between children with and without DKAe. We found a significant association between slower CV and MCV of the eyes on admission and the presence of encephalopathy. Only the encephalopathic group showed statistically significant changes in CV and MCV between the first and second readings, likely related to the resolution of encephalopathy and clinical improvement. There was no significant difference in CV and MCV between groups at T1, suggesting that the slowing of the CV and MCV resolves and corrects to normal values once the encephalopathy subsides. The times for DKA resolution, while approximate, were similar between both groups. In addition, the diagnostic performance of CV and MCV for the eyes at admission was robust, with AUCs of 0.864 and 0.845, respectively, indicating strong discriminatory ability.

Several studies have been conducted to establish reference values for pupillometry measurements in pediatric patients, and one study reported a mean CV of 3.70 mm/s (range: 2.21-5.18 mm/s, mean ± 2 SD) and a mean MCV of 5.02 mm/s (range: 3.23-6.81 mm/s) [19]. In our study, encephalopathic patients had a mean CV of 2.17 mm/s (range: 1.60-2.74 mm/s) and a mean MCV of 3.32 mm/s (range: 2.28-4.36 mm/s). In contrast, non-encephalopathic patients showed a mean CV of 3.21 mm/s (range: 2.79-3.63 mm/s) and a mean MCV of 5.01 mm/s (range: 4.37-5.66 mm/s). The values for the non-encephalopathic group were consistent with the published reference ranges, while the encephalopathic group exhibited slower pupillometry measurements, supporting our hypothesis.

Our study has several limitations. First, the small sample size limits statistical power and the generalizability of our findings. As a single-center study with 21 patients, our results may not be representative of broader, more diverse populations. Second, the retrospective design inherently introduces limitations, including dependence on documentation accuracy and lack of standardized data collection. The determination of encephalopathy was based on subjective clinical assessments documented in the medical record, without a validated scale or quantification of severity, introducing potential diagnostic variability. Visual assessments of pupil reactivity were inconsistently documented (e.g., described as "brisk" or "sluggish"), making direct comparisons with pupillometry data impossible. Consistent bedside documentation of penlight-based pupillary reflex assessments would have been valuable for comparison and should be prioritized in future studies. Moreover, this variability in subjective assessments and documentation practices may have impacted the interpretation of encephalopathy diagnosis and limited the clinical utility of pupillometry in standard PICU settings, where heterogeneity in bedside examination techniques can confound correlations between clinical examination and objective measurements. Future prospective studies should incorporate standardized, detailed documentation of visual pupillary assessments and use validated tools such as the GCS to help quantify encephalopathy severity. Collectively, these limitations underscore the need for larger, multicenter studies to validate our findings and further define the clinical role of quantitative pupillometry in pediatric DKA.

## Conclusions

Our study suggests that quantitative pupillometry, specifically the measurement of CV and MCV, may serve as a valuable, noninvasive tool for detecting and monitoring DKAe in pediatric patients. The observed slowing of CV and MCV at admission in encephalopathic patients, and their subsequent normalization with clinical recovery, supports the potential role of pupillometry as an objective marker of neurological status in DKA. Given its reproducibility and reduced interobserver variability compared to traditional clinical assessments, pupillometry could enhance the accuracy and sensitivity of neurological monitoring in the PICU. Incorporating this technology into standard care protocols may facilitate earlier recognition of cerebral involvement, prompt timely interventions, and ultimately improve outcomes in children with DKA. Furthermore, abnormalities in the pupillary light reflex can indicate autonomic nervous system dysfunction, raising intriguing questions about the impact of the autonomic system in the pathobiology of DKA. This novel perspective underscores the need for further research to better elucidate these mechanisms.
